# scHybridBERT: integrating gene regulation and cell graph for spatiotemporal dynamics in single-cell clustering

**DOI:** 10.1093/bib/bbae018

**Published:** 2024-02-11

**Authors:** Zhang Wei, Wu Chenjun, Xing Feiyang, Jiang Mingfeng, Zhang Yixuan, Liu Qi, Shi Zhuoxing, Dai Qi

**Affiliations:** Zhejiang Sci-Tech University, 310028, Hangzhou, China; Zhejiang Sci-Tech University, 310028, Hangzhou, China; Translational Medical Center for Stem Cell Therapy and Institute for Regenerative Medicine, Shanghai East Hospital, Frontier Science Center for Stem Cell Research, Bioinformatics Department, School of Life Sciences and Technology, Tongji University, 200092, Shanghai, China; Zhejiang Sci-Tech University, 310028, Hangzhou, China; Zhejiang Sci-Tech University, 310028, Hangzhou, China; Translational Medical Center for Stem Cell Therapy and Institute for Regenerative Medicine, Shanghai East Hospital, Frontier Science Center for Stem Cell Research, Bioinformatics Department, School of Life Sciences and Technology, Tongji University, 200092, Shanghai, China; State Key Laboratory of Ophthalmology, Zhongshan Ophthalmic Center, Sun Yat-Sen University, Guangdong Provincial Key Laboratory of Ophthalmology and Visual Science, 510060, Guangzhou, China; Zhejiang Sci-Tech University, 310028, Hangzhou, China

**Keywords:** multi-view modeling, BERT architecture, spatiotemporal embedding, cell graphs, graph attention networks

## Abstract

Graph learning models have received increasing attention in the computational analysis of single-cell RNA sequencing (scRNA-seq) data. Compared with conventional deep neural networks, graph neural networks and language models have exhibited superior performance by extracting graph-structured data from raw gene count matrices. Established deep neural network-based clustering approaches generally focus on temporal expression patterns while ignoring inherent interactions at gene-level as well as cell-level, which could be regarded as spatial dynamics in single-cell data. Both gene–gene and cell–cell interactions are able to boost the performance of cell type detection, under the framework of multi-view modeling. In this study, spatiotemporal embedding and cell graphs are extracted to capture spatial dynamics at the molecular level. In order to enhance the accuracy of cell type detection, this study proposes the scHybridBERT architecture to conduct multi-view modeling of scRNA-seq data using extracted spatiotemporal patterns. In this scHybridBERT method, graph learning models are employed to deal with cell graphs and the Performer model employs spatiotemporal embeddings. Experimental outcomes about benchmark scRNA-seq datasets indicate that the proposed scHybridBERT method is able to enhance the accuracy of single-cell clustering tasks by integrating spatiotemporal embeddings and cell graphs.

## INTRODUCTION

Single-cell RNA sequencing (scRNA-seq) technology has provided a powerful way to explore cellular heterogeneity and identify cell subclusters with high resolution. Compared with traditional bulk RNA sequencing, scRNA-seq data allow for the analysis of gene expression at the individual cell level, providing a more accurate representation of the transcriptomic profiles of different cell types and subclusters. In particular, scRNA-seq data clustering has been regarded as a critical step in identifying and characterizing these sub-clusters, as it enables the detection of distinct transcriptomic profiles that may correspond to different cell types or states. As cell type detection is essential to inform the selection of target molecules and pathways, deep neural networks have been widely employed in downstream tasks such as cell type annotation and cell clustering [1, 2].

Deep learning methods developed for scRNA-seq data have been used to reveal the molecular and regulatory mechanisms that drive complex biological processes, including disease progression and cell differentiation. For human beings, the aging process is widely believed to be associated with changes in the distribution of cell types and gene expression patterns. Utilizing scRNA-seq data modeling allows for the identification of key factors in the aging process and the discovery of potential regulatory mechanisms. Specific diseases, including neurodegenerative disorders, are supposed to have a close association with single-cell transcriptomics [3, 4]. In order to obtain biologically meaningful patterns, multi-omics data platform such as AMP-AD and AMP-PD databases have been constructed to identify biomarkers as well as to investigate the molecular mechanisms that are associated with neurodegenerative disorders. To deal with heterogeneous datasets, multi-modal learning approaches and feature fusion technology make it feasible to identify molecular biomarkers and detect disease subtypes from genetics and transcriptomics [5–9].

However, accurate clustering of scRNA-seq data is still challenged due to high levels of biological noise and dropout events, which lead to false zero counts. For single-cell clustering tasks, plenty of deep learning-based approaches have been proposed to detect cell sub-clusters from gene expression data [10–12]. Design of network structures and learning objectives may take into consideration the characteristics of single-cell data, aiming to achieve high accuracy and reliability in downstream tasks [13, 14]. These clustering methods involve feature selection and dimensionality reduction to mitigate the impact of dropout events and other biological noise in single-cell data [15–17]. Owing to the strong capability to mine signal patterns, complicated deep learning models including generative models and transformers are also employed in the analysis and modeling of single-cell data. However, existing deep learning models generally focus on temporal patterns underlying single-cell RNA sequencing data and ignore spatial patterns to some degree. Graph-structured data extracted from gene expression data have been regarded as typical temporal patterns.

Deep learning models have been widely employed in analyzing single-cell data since deep neural networks have exhibited excellent ability in various downstream tasks including cell type annotation as well as network inference [18]. Among these deep learning models, the scDeepCluster algorithm utilized deep-embedded clustering to learn both feature representations by explicitly modeling the generation of scRNA-seq data [19]. As another cell clustering method, SIMLR learns proper weights for multi-kernels for the gene expression matrix. This SIMLR method obtains an appropriate cell-to-cell similarity metric from the input single-cell data and constructs a symmetric similarity matrix. The unsupervised DESC method also utilizes deep embedding [20] but is equipped with an iterative self-learning paradigm that aims to overcome the challenges of batch effects. Not limited to deep neural networks, large language models also have played a role in the computational analysis of single-cell transcriptomics. Fast advancements in natural language processing have been propelled by the bidirectional encoder representations from transformers (BERT) model [21]. One application of large-scale language models in single-cell data analysis is the single-cell BERT model [22], which exploits gene expression data to unveil the transcriptional patterns of cells.

Established deep learning-based single-cell data analysis approaches mainly focus on a single perspective, such as abnormal expression patterns of marker genes. This practice often ignores inherent functional interactions between cells or genes. Such directed interactions, which take the form of graph-structured data, may be useful to boost the accuracy of downstream tasks including single-cell clustering [23]. In previous works, topological features have been regarded as a kind of deep-level feature underlying RNA-seq data [24, 25]. To deal with graph-structured features, various graph neural networks-based methods have been developed to capture interactions between cells as well as genes [26–29]. In addition, graph neural networks have shown superior performance in dealing with graph-structured features. One possible explanation is that graph-structured features provide latent cell–cell and gene–gene interactions to boost the model performance in downstream tasks. Cell–cell similarity or interaction has been regarded as valuable information in downstream tasks. For scRNA-Seq data, multi-modality learning has become a promising solution in integrating temporal patterns and graph-structured features.

This study proposes a multi-view modeling based scHybridBERT framework to identify cell types with spatiotemporal embeddings and cell graphs. This multi-view modeling structure consists of spatial and temporal dynamics at the molecular level. In order to conduct multi-view modeling, both cell graphs and spatial embedding capture topological features of regulatory systems, while gene and expression embedding are regarded as temporal patterns. In order to extract dynamics about gene–gene interactions, this scHybridBERT architecture computes spatial embedding by inferring gene co-expression networks from scRNA-seq data. Meanwhile, cell graphs are constructed using a novel exponential Manhattan (Exp-Mah) similarity metric and are employed by graph attention networks (GATs). Subsequently, an adaptive multilayer perceptron (MLP)-based fusion strategy was applied to integrate hybrid data modalities including spatiotemporal embedding and graph-structured data. Experiments of multiple scRNA-seq datasets with cell-type labels are conducted to illustrate the feasibility and effectiveness of the scHybridBERT method.

## BACKGROUND OF BERT MODEL

Established works illustrate that BERT architecture has shown superior performance in the computational analysis of scRNA-Seq data. In the BERT architecture, transformer models are able to inspect dependencies across the entire dataset, thus learning a global context. By masking and training on unlabelled scRNA-seq data with a self-supervised style, the Transformers model and its derivatives explore dependencies across the entire dataset, thus capturing global sequences and detecting nuanced dependencies. Pre-training and fine-tuning mechanisms have played a crucial role in the conventional scBERT model.

### Preprocessing of raw scRNA-seq data

Since raw data may contain biological noise and technical artifacts, feature engineering of the raw data matrix is essential. In general, non-zero expression values in the raw count matrix usually only account for $\sim $10$\%$. Thus, gene expression values are transformed to ensure normality. At the same time, the expression of all genes was preserved in order to obtain a comprehensive viewpoint about gene graphs and cell graphs for subsequent analysis.

As token embedding and position embedding have been considered in the BERT architecture, it is necessary to fully utilize the characteristics of gene sequences during the analysis of scRNA-seq data. The token embedding is a discrete variable, whereas the raw expression input is a continuous variable standing for the expression of a gene with biological noise. The bag-of-words technology has been employed to bin the expression of genes which could be regarded as the gene transcript frequency in the cell.

For scRNA-seq data, gene embedding and expression embedding are computed as inputs for the pre-trained language model. Pre-training mechanism adopted by scBERT can only analyze the genetic features learned during pre-training. If genes in given scRNA-seq datasets are not included in the pre-training parameters, they were directly removed, thus affecting model accuracy and generalization performance. In this case, scHybridBERT proposes an end-to-end framework, which has strong generalization performance.

### Gene embedding

Gene embedding was obtained by the gene2vec algorithm to represent gene identity which was viewed as relative embedding to capture the semantic similarity from the aspect of general co-expression. Co-expressed genes retain closer representations, and distributed representations of genes are useful for capturing gene–gene relations.

This section draws on the ideas of word2vec to learn the continuous vector representation of gene expression so that each segment in the gene sequence is mapped to a vector representation in a continuous vector space. These vectors have semantic meaning, making similar gene fragments closer in the vector space. The objective function of the gene2vec algorithm is defined as follows: 


(1) \begin{align*}& L=-\sum_{w\in D}\sum_{c\in{C_{w}}}\log{P(c|w),}\end{align*}

where $D$ is a text corpus containing all genes and $C_ W $is a context set related to gene $w$. The gene2vec algorithm employs the skip-gram mechanism to learn word vectors. Given a gene $w$, the goal is to maximize the conditional probability $P(c|w)$ of the context gene $c$ associated with the gene. Specifically, this conditional probability can be calculated as follows: 


(2) \begin{align*}& P(c|w)=\frac{e^{v_{c} \cdot v_{w}}}{\sum_{c^{\prime} \in D} e^{v_{c^{\prime}} \cdot v_{w}}},\end{align*}

where $v_{W}$ and $v_{C}$ are the vector representation of genes $w$ and $c$, respectively. Specifically, the gene2vec algorithm was used to produce gene embeddings that capture the semantic similarities between genes. These gene embeddings, along with the discretized expression embeddings, have played the role of temporal embedding of the Performer model, allowing for the extraction of temporal information at the molecular level. Gene embedding denotes gene identity from gene2vec falling into the first bin, while the expression embedding is associated with gene expression falling into the second bin and being transformed in the same direction as gene embedding.

### Temporal expression embedding

In addition to gene embedding, the transcription level of each gene provides valuable information as continuous variables. The difference between gene embedding and expression embedding lies in the different views of scRNA-seq data. Similar to language models, the expression level of genes has been considered as a similar occurrence in biological systems. Therefore, the term-frequency-analysis method was employed to discretize the continuous expression variables by binning, thus obtaining 200-dimensional vectors that serve as token embeddings for model training.

Gene embedding refers to the method of converting gene sequence information into a vector representation, with the main purpose of describing the similarities and differences between genes. Different from expression embedding, it is a method of converting gene expression profile data into a vector representation, intending to describe the expression of genes under different conditions.

### Performer model

The performer can be used to overcome the high dimension and noise of the data and reveal the underlying graph structure of single-cell transcriptomic data. As a deep neural network model that introduces the self-attention mechanism, the Performer model owns the advantage of the attention mechanism with reduced space complexity. Performer is regarded as an extension of the Transformer and is also based on the self-attention mechanism. Such mechanisms have the advantage of achieving similar effects to attention mechanisms with limited spatial complexity and high efficiency in capturing long-distance dependencies with fewer computational resources during processing sequence data. This enables Performer to have better performance and scalability when processing large-scale single-cell sequencing data. In the Performer, the self-attention mechanism is represented by the following formula: 


(3) \begin{align*}& Attention(Q,K,V)=D^{-1}(QK^{T})V,D=diag(QK^{T}1_{L}),\end{align*}

where $Q$ is the query vector, $K$ and $V$ denote the key and value vector, respectively, and $d_{k}$ represents the dimensionality of the query vector. This formula can be used to calculate the similarity between each cell and other cells, thus grouping them into different cell sub-clusters. By using low-rank approximation and random feature techniques, Performer reduces the computational complexity of the self-attention mechanism from $O(n^{2})$ to $O(n \log n)$, where $n$ is the length of the sequence. This indicates that the Performer has improved the efficiency in computing attention matrices and has exhibited enhanced capability to handle longer sequences than the standard transformer model.

## OVERVIEW OF THE SCHYBRIDBERT FRAMEWORK

**Figure 1:**
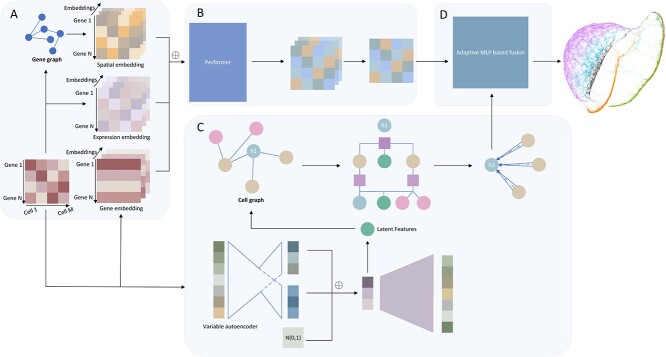
The flowchart of multi-view scHybridBERT method. This multi-view architecture consists of spatial and temporal patterns underlying single-cell transcriptomics. In addition to temporal embedding, spatial embeddings and cell graphs are constructed to capture gene–gene and cell–cell interactions underlying scRNA-Seq data, respectively. After inputting three types of embeddings into the performer, spatiotemporal patterns extracted from single-cell data are aggregated by the adaptive MLP mechanism. The symbol $\oplus $ represents an element-wise addition operation.

Motivated by language models, the scHybridBERT method is proposed to conduct multi-view modeling for scRNA-seq data, thus obtaining a more comprehensive model. Heterogeneous information including spatiotemporal dynamics and cell graphs are employed by the Performer model and graph neural networks, respectively. Spatiotemporal embedding and graph-structured data are subsequently integrated by an adaptive MLP-based fusion strategy. Performer model aims to deal with long single-cell RNA sequences, capturing global information, while graph neural networks discover complex clustering structures and compensate for the lack of isolated sample points. The basic diagram of the scHybridBERT method is demonstrated by Figure 1.

In this diagram, global topological information of molecular systems was contained by gene regulatory networks and co-expression networks. Such topological information has not yet been explicitly incorporated into previous single-cell clustering methods. Spatial and temporal embeddings are combined by element-wise addition. while the Exp-Mah distance metric that combines Manhattan distance and correlation coefficient is applied to learn deep-level features about cell-level interactions.

In another pipeline of this multi-view modeling structure, latent features that are computed by variable autoencoder are used to train GATs. Afterward, heterogeneous data modalities are integrated by the adaptive MLP-based fusion strategy.

### Construction of spatial embedding

Among spatiotemporal embedding extracted from scRNA-Seq data, gene co-expression networks were computed as spatial embedding to capture gene–gene interactions at the molecular level. The proposed scHybridBERT method infers gene co-expression networks from single-cell RNA sequencing data and employs neighborhood information to obtain gene–gene interactions. Extracted spatial embeddings provide the topological perspective in computational modeling about RNA-seq data. Co-expression relationship between genes is defined as Eq (4) 


(4) \begin{align*}& \text{cos}(\theta) = \frac{\mathbf{x} \cdot \mathbf{y}}{\|\mathbf{x}\| \|\mathbf{y}\|} = \frac{\sum\limits_{i=1}^{n} x_{i} y_{i}}{\sqrt{\sum\limits_{i=1}^{n} x_{i}^{2}} \sqrt{\sum\limits_{i=1}^{n} y_{i}^{2}}},\end{align*}

where $\mathbf{x}$ and $\mathbf{y}$ represent the vector expression of two genes, $x_{i}$ and $y_{i}$ represent the value of the $i$th element in the vector and $n$ represents the dimension of feature vector. In this work, the node2vec algorithm has been used to capture the gene–gene relations to embed patterns [30]. Given the current vertex $v$, the probability description formula for accessing the next vertex $x$ is described as Eq (5) 


(5) \begin{align*}& P(c_{i}=x|c_{i-1}=v)=\left\{ \begin{aligned} \frac{\pi_{vx}}{Z} &, & if (v,x) \in E, \\ 0 &, & \text{otherwise} \end{aligned}, \right.\end{align*}

where $\pi _{vx}$ is the non-normalized transition probability between vertices $v$ and $x$, and $Z$ is the normalization constant. Node2vec introduces two hyperparameters $p$ and $q$ to control the strategy of random walk, assuming that the current random walk passes through edges $(t, v)$ and reaches vertex $v$. Set $\pi _{vx} = \alpha _{pq}\cdot w_{vx}$, where $w_{vx}$ denotes the edge weight between vertices $v$ and $x$, the definition of $\alpha _{pq}$ is given as Eq (6) 


(6) \begin{align*}& \alpha(t,x)=\left\{ \begin{aligned} \frac{1}{p} &, & if\ d_{tx} =0, \\ 1 &, & if\ d_{tx} =1 \\ \frac{1}{q} &, & if\ d_{tx} =2, \\ \end{aligned}\right.\end{align*}

where $d_{tx}$ denotes the shortest path distance between vertex $t$ and $x$. As a parameter, the variable $p$ controls the probability of repeatedly accessing vertices that have just been accessed. If $p$ is high, the probability of accessing vertices that have just been accessed will decrease. The parameter q controls whether the walk is outward or inward. If $q>1$, the random walk tends to access vertices that are close to $t$, and vice versa, it tends to access vertices that are far away from $t$. As a node embedding algorithm, increasing $p$ and decreasing $q$ is able to capture co-expression information between genes while improving algorithm efficiency.

### Construction of cell graphs

Single-cell RNA sequencing technology can generate a huge amount of gene expression data from individual cells. In this technology, the raw gene count matrix usually has a high dimension, while the number of cells is relatively limited, making it difficult to analyze the expression matrix and uncover cell relationships directly. To alleviate this problem, the VAE model was used to reduce the dimension of the gene expression matrix and obtain low-dimensional features. These expression-related features are represented as the embedding of encoded cells. With embedded expressions, the scHybridBERT framework further constructs cell graphs to capture cell–cell interactions, with the purpose of integrating spatial dynamics in cell clustering tasks.

The encoder part of the VAE model contains two fully connected layers, $fc_{1}$ and $fc_{2}$, along with a ReLU activation function. The encoder of the VAE model is defined as follows: 


(7) \begin{align*}& \begin{aligned} \mu &= fc_{21}(relu(fc_{1}(x))\\ \log\sigma^{2} &= fc_{22}(relu(fc_{1}(x)), \end{aligned}\end{align*}

where $\mu $ and $\log \sigma ^{2}$ represent the posterior mean and log variance, respectively. The latent variable zis obtained using the parameterization trick, shown by Eq (8) 


(8) \begin{align*}& \begin{aligned} z=\mu+\sigma\epsilon, \quad \epsilon\sim \mathcal{N}(0,I). \end{aligned}\end{align*}

The decoder part of the VAE model consists of two fully connected layers, denoted by $fc_{3}$ and $fc_{4}$, along with a sigmoid activation function. This is used to decode the latent variable $z$ back into a reconstruction of the original input $x$, denoted as Eq (9) 


(9) \begin{align*}& \begin{aligned} h_{3}&=relu(fc_{3}(z))\\ x^{^{\prime}}&=sigmoid(fc_{4}(h_{3})). \end{aligned}\end{align*}

The encoder block of the VAE model maps input data into the latent space, and the decoder block reconstructs the input from latent representations. Reconstruction loss of the VAE model is computed according to Eq (10) 


(10) \begin{align*}& L_{MSE}=\frac{1}{N}\sum_{i=1}^{N} \left\Vert \mathbf{x}_{i} - f(g(\mathbf{x}_{i})) \right\Vert^{2},\end{align*}

where $f(g(\mathbf{x}_{i}))$ represents the output result of the AE model and $\mathbf{x}_{i}$ is a sequence of the gene expression value. $\left \Vert \mathbf{x}_{i} - f(g(\mathbf{x}_{i})) \right \Vert ^{2}$ indicates the 2-norm of sequences and can be further interpreted as $\sum _{j=1}^{M} |\delta _{j}|$, where $\delta _{j}$ is the $j$th value of a certain cell, which is the expression amount of one of its genes.

This VAE model is trained using a reconstruction loss function, including Mean Squared Error (MSE) and Mean Absolute Error. Only reconstruction loss may not guarantee the continuity and interpretability of generated latent representations. To control the distributions of latent representations, the Kullback–Leibler (KL) divergence was used as a regularization term to constrain the distributions of latent representations. In this study, KL divergence is applied to measure the difference between learned latent representation and prior distributions, defined by Eq (11) 


(11) \begin{align*}& L_{KL} = \frac{1}{2}\sum_{i=1}^{d}(1+\log(\sigma_{i}^{2})-\mu_{i}^{2}-\sigma_{i}^{2}),\end{align*}

where $\mu $ and $\sigma $ represent the mean and standard deviation of latent representations obtained by the autoencoders, and $d$ represents the dimensions of latent representations. The goal of this loss function is to constrain the distribution of latent representations to standard normal distribution. The final loss function consists of two components, denoted by Eq (12) 


(12) \begin{align*}& L = \alpha L_{MSE} + (1-\alpha) L_{KL},\end{align*}

where $L_{\text{MSE}}$ represents the reconstruction loss function, $\lambda $, and $\mu $ represents the regularization parameter, which is used to balance the weight of the two loss functions. Low-dimensional representations of gene expression corresponding to cells were generated after training the VAE model. Therefore, similarities and differences between cells were extracted and employed, with significantly reduced computational complexity.

### Exp-Mah metric in capturing cell–cell interactions

In the previous study, the Euclidean distance metric was used to measure the functional similarity between two samples by calculating the Euclidean distance between them. However, Euclidean distance was affected by the curse of dimension in high-dimensional data. This indicates that Euclidean distance is highly sensitive to outliers, which were commonly observed in gene expression matrices. To alleviate this problem, the scHybridBERT architecture adopts the novel exponential Manhattan (Exp-Mah) distance which comprehensively considers the correlation values of gene expression levels.

This Manhattan distance $d_{M}(\vec{x},\vec{y})$ is defined as follows: 


(13) \begin{align*}& \begin{aligned} d_{M}(\vec{x},\vec{y}) &=\sum_{i=1}^{N} |x_{i}-y_{i}|, \end{aligned}\end{align*}

where $\vec{x}$ and $\vec{y}$ represent the n-dimensional gene vectors of two different cells, respectively, $x_{i}$ and $y_{i}$ denote the values of the $i$th gene on the two cells and $N$ represents the number of genes in the cell. With the Exp-Mah distance, two vectors were projected onto a Gaussian plane and the distance was calculated. After dimensional reduction, the features of gene sequences become independent and contain positive and negative values. The correlation coefficients can comprehensively consider positive and negative features and are calculated as Eq (14) 


(14) \begin{align*}& \begin{aligned} R_{cor}(\vec{x},\vec{y}) &= \frac{\sum_{i=1}^{n}(x_{i}-\bar{x})(y_{i}-\bar{y})}{\sqrt{\sum_{i=1}^{n}(x_{i}-\bar{x})^{2}} \sqrt{\sum_{i=1}^{n}(y_{i}-\bar{y})^{2}}}, \end{aligned}\end{align*}

where the n-dimensional gene vectors $\vec{x}$ and $\vec{y}$ are obtained from two different cells, $x_{i}$ and $y_{i}$ represent the values of the $i$th gene on the two cells, $\bar{x}$ and $\bar{y}$ are the mean values of the two vectors and $N$ represents the number of genes in the cell. Therefore, the formula for measuring cell similarity $Sim(\vec{x},\vec{y})$ is defined as Eq (15) 


(15) \begin{align*}& \begin{aligned} sim(\vec{x},\vec{y}) &= R_{cor}e^{\alpha d_{M}}, \end{aligned}\end{align*}

where $\alpha $ is a hyperparameter that adjusts the weights of two measurement metrics. Exponential function grows rapidly when the independent variable increases. Direct linear combinations may lead to an inconsistent range of similarity scores. Employment of the exponential function in Exp-Mah distance can combine the two together to unify the range of similarity scores.

Cell graphs are constructed using the KNN algorithm, where each node represents a single cell and the edges between nodes represent similarities or affiliations between cells. Constructing cell graphs requires setting the number of neighbors, which is related to the scale of cell–cell interactions captured in the graph. Each cell node finds its neighbor cells within $K$ shortest distances and creates edges between them. Therefore, the value of $K$ affects the density and complexity of the constructed cell graph. Large $K$ values will lead to denser edges in cell graphs. Construction of the cell graphs reveals important cell interaction patterns in single-cell RNA-seq data. 

### Pruning of cell graphs

Graph learning methods act as the core part of the proposed scHybridBERT model and employ cell graphs to detect cell subpopulations. Graph neural networks have been used for node-level representation learning on large graphs. One of the key components of graph neural networks is the aggregation process, which combines information from a node’s neighbors to update its representation. The aggregation process in graph learning can be broken down into three main steps: sampling, message passing and aggregation. In the study, Graph neural networks are used as the aggregation method, and the aggregation function is defined as follows: 


(16) \begin{align*}& h_{\mathrm{agg}}^{(i)} = \mathrm{ReLU} \left( \sum_{j \in \mathcal{N}(i)} \frac{1}{c_{ij}} W h_{\mathrm{in}}^{(j)} \right),\end{align*}

where $h_{\text{in}}$ represents the input representation of a node, $h_{\text{agg}}$ denotes the aggregated representation, $W$ is a learnable weight matrix, $\mathcal{N} (i)$ represents the set of neighboring nodes of node $i $ and $c_{ij}$ is a normalization factor defined by Eq (17) 


(17) \begin{align*}& c_{ij} = \sqrt{d_{i}} \cdot \sqrt{d_{j}},\end{align*}

where $d_{i}$ and $d_{j}$ are the degree of nodes $i$ and $j$, respectively. The aggregation function is regarded as a normalized weighted sum of the input representations of a node’s neighbors. The normalization factor $c_{ij}$ accounts for the varying degrees of the nodes and ensures that the aggregation is not biased toward nodes with higher degrees. The rectified linear unit activation function $ReLU$ is applied element-wise to the aggregated representation to introduce non-linearity.

### Training of GAT using cell graphs

Compared with conventional graph neural networks, GATs have enhanced capability to deal with large-scale graphs, especially for cell graphs involving thousands of nodes. An attention mechanism was employed to learn the weights of neighboring nodes and to obtain the expression of the nodes themselves. This mechanism can automatically allocate the weights of neighboring nodes and capture the relationships between nodes. In this case, the GAT model captures the relations between nodes in the graph, enabling end-to-end learning on graph-structured data.

Graph-structured data were represented as a graph $G = (V, E)$, where $V$ is the set of nodes and $E$ is the set of edges. Typically, an adjacency matrix $A \in \mathbb{R}^{N \times N}$ is used to represent the topology of the graph, where $N$ is the number of nodes, and $A_{ij}$ indicates whether there is an edge between node $i$ and node $j$. Moreover, we can have a feature matrix $X \in \mathbb{R}^{N \times F}$, where $F$ is the number of features for each node.

The basic building block of GAT is the graph attention Layer where the input feature matrix $X$ was linearly transformed into updated feature matrix $H \in \mathbb{R}^{N \times F^{^{\prime}}}$, where $F^{^{\prime}}$ is the number of output features. This can be achieved by matrix multiplication with learnable weight matrix $W \in \mathbb{R}^{F \times F^{^{\prime}}}$, denoted as Eq (18) 


(18) \begin{align*}& H = XW,\end{align*}

where $W$ denotes a learnable weight matrix. The affinity score between node $i$ and node $j$ is calculated to determine the attention weights between nodes. This can be done by taking the inner products of heterogeneous features and applying a nonlinear activation function. This nonlinear LeakyReLU activation function in the GAT model takes the form of Eq (19) 


(19) \begin{align*}& e_{ij} = \text{LeakyReLU}(\boldsymbol{a}^{T} [H_{i} \parallel H_{j}]),\end{align*}

where $\parallel $ denotes cascade operation between vectors, $\boldsymbol{a} \in \mathbb{R}^{2F^{\prime}}$ is a learnable weight vector and $H_{i}$ and $H_{j}$ represent the feature vectors of nodes $i$ and $j$, respectively. To learn the attention weights of node $i$ with respect to its neighboring nodes, we normalize the affinity scores of nodes with the softmax function. The normalized attention weight $\alpha _{ij}$ is computed according to Eq (20) 


(20) \begin{align*}& \alpha_{ij} = \frac{\exp(e_{ij})}{\sum_{k \in \mathcal{N}(i)} \exp(e_{ik})},\end{align*}

where $\mathcal{N}(i)$ denotes the neighborhood of node $i$. In order to update the feature vector for node $i$, attention weights are used to calculate the weighted sum of the features of neighboring nodes, shown by Eq (21) 


(21) \begin{align*}& H_{i}^{^{\prime}} = \sum_{j \in \mathcal{N}(i)} \alpha_{ij} H_{j},\end{align*}

where $H_{i}^{^{\prime}}$ is the updated feature vector for node $i$. To enhance the model’s predictive capacity, the GAT model employs multi-head attention, which consists of multiple independent attention mechanisms. The output feature vectors from each attention head are concatenated or averaged to obtain the final output. The output of a multi-head attention layer can be represented by Eq (22) 


(22) \begin{align*}& H^{^{\prime}} = \frac{1}{K} \sum_{k=1}^{K} \text{GAT}_{k}(H),\end{align*}

where $\text{GAT}_{k}$ represents the $k$th attention head, and $K$ is the number of attention heads. By stacking multiple graph attention layers, GAT models are able to learn complex representations of cell graphs. By introducing the attention mechanism, GAT are able to effectively capture the relationships between nodes and perform end-to-end learning. The pseudo-code of the scHybridBERT method is described as Algorithm 1.


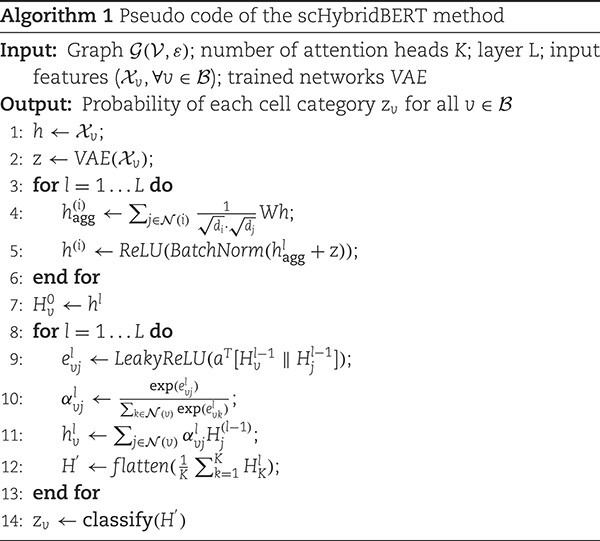


In Algorithm 1, step 5 aims to compute $h^{(i)}$ which corresponds to latent variables in Figure 1, while step 9 of calculating $e_{vj}^{l}$ is the process of training GATs using cell graphs.

### Adaptive MLP-based fusion strategy

In this study, heterogeneous information including spatiotemporal embeddings and cell graphs have been employed to provide the spatial viewpoint in cell clustering. This scHybridBERT method aims to conduct multi-view modeling of scRNA-seq data by fusing spatial and temporal dynamics, which correspond to different data modalities. Heterogeneous data modalities $P$, which represent the prediction probabilities of two information sources, are defined as Eq (23) 


(23) \begin{align*}& P = W_{1}A+W_{2}B,\end{align*}

where $W_{1}$ and $W_{2}$ denote adaptive weights corresponding to the performance of the Performer and graph neural network, respectively. The matrices $A$ and $B$ represent the extracted information from the performer and graph neural network, respectively.

Based on the prior knowledge obtained during model training, the model performance metrics of the Performer and GAT can be used as the weight distribution configuration reliability, and the assignment of weights is determined by Eq (24) 


(24) \begin{align*}& \begin{aligned} W_{1} = \frac{Perf_{A}}{Perf_{A}+Perf_{B}} \\ W_{2} = \frac{Perf_{B}}{Perf_{A}+Perf_{B}}. \end{aligned}\end{align*}

In Eq (24), $Perf_{A}$ and $Perf_{B}$ represent the predictions obtained by the Performer and graph neural network, respectively. When the two data modalities produce different types of errors or biases, the fusion strategy can balance errors and enhance the model’s performance.

## EXPERIMENTAL RESULTS AND ANALYSIS

In single-cell clustering tasks, the experiment section investigates the characteristics of the scHybridBERT architecture in cell-type detection from multiple perspectives. Extraction and employment of cell graphs will be discussed in detail. During the process of constructing cell graphs, multiple distance metrics are compared to illustrate the advantage of the Exp-Mah metric used in the scHybridBERT architecture. Meanwhile, the function of spatial embedding will also be discussed. Eventually, distributions of cell subpopulations as well as performance comparisons are conducted in multiple scRNA-seq datasets, thus providing a comprehensive evaluation of single-cell clustering task. Benchmark scRNA-seq datasets are described in Table 1.

**Table 1:** Description of benchmark scRNA-seq datasets with cell labels

Datasets	Cell types	Cell number	gene number
Zeisel	9	3005	19 972
Mouse	16	2100	20 670
Klein	4	2717	24 047
AD-brain	8	13 214	10 852
PBMC-Kang-A	8	11 432	14 504
PBMC-Kang-B	8	12 261	14 473
PBMC-Kang-C	8	11 989	14 222
PBMC-Zheng4k	8	4340	33 694
PBMC-Zheng7k	11	2843	9837

### Exp-Mah metrics in constructing cell graphs

**Figure 2:**
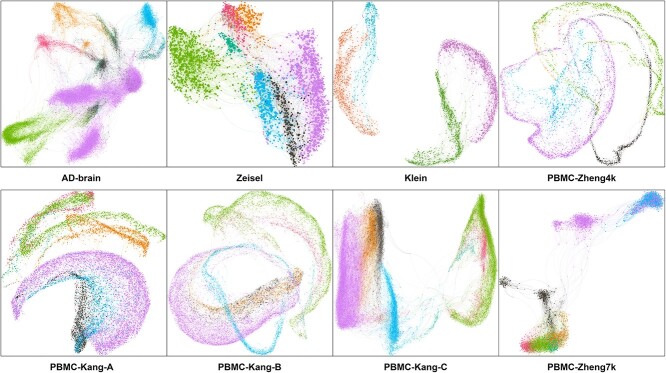
Visualization of cell graphs that are extracted single-cell transcriptomics data. After extracting latent features from variational autoencoders, an exponential Manhattan (Exp-Mah) distance is used to capture cell–cell communication and construct cell graphs. The colors of each node are represented by their true categories.

Extraction and employment of cell graphs from scRNA-seq data is a crucial step in the scHybridBERT framework. Such cell graphs reflect the functional similarities between cell pairs and provide valuable information to enhance the clustering performance. As Euclidean distance may not be the optimal choice for single-cell expression data, this study designs an efficient metric to construct cell graphs and validate the effectiveness of this metric over other candidate options. For eight groups of scRNA-seq data, cell graphs captured by graph neural networks and are visualized as Figure 2.

In Figure 2, cell graphs that are extracted from three types of PBMC data exhibited significant inconsistency, demonstrating various cell–cell communication patterns. Such graph information has the potential to boost the clustering accuracy. With Euclidean and exp-Mah metrics, replication experiments were conducted and evaluation metrics were computed during the construction of cell graphs.

**Figure 3:**
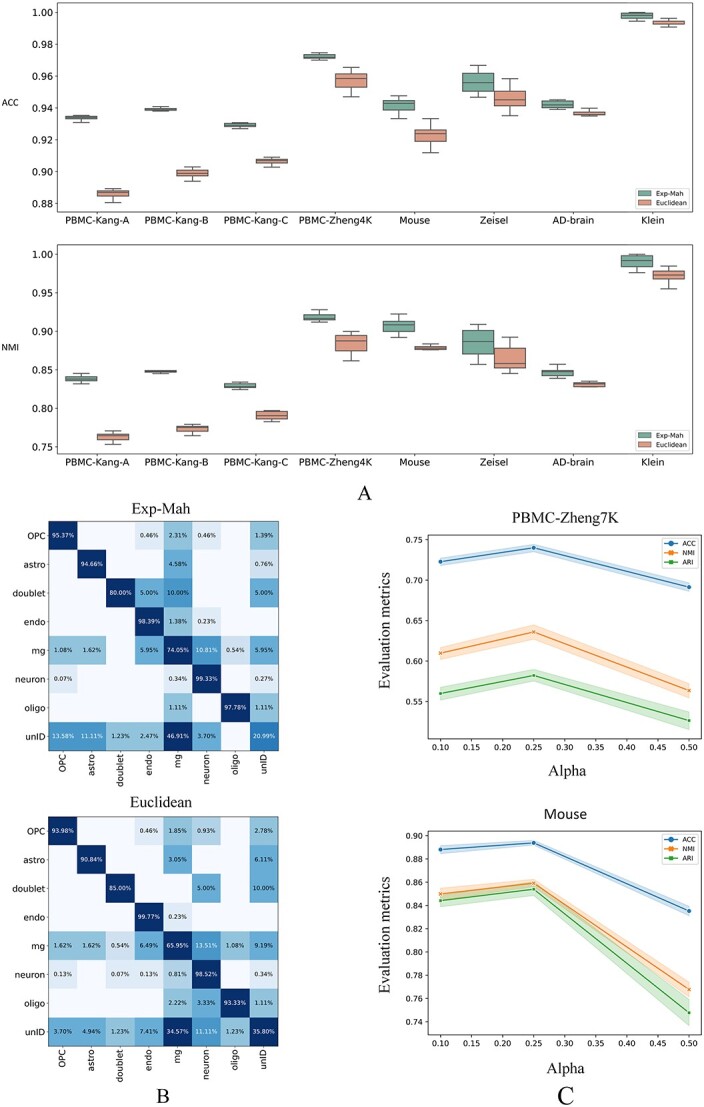
Comparison of Euclidean and Exp-Mah distance metrics during cell graph construction. Subgraph **A** demonstrates the clustering indexes obtained by Euclidean and Exp-Mah metrics on eight labeled scRNA-seq datasets. The box plots in (**A**) describe the median, interquartile range and extreme values of single-cell clustering results. Subgraph **B** demonstrates the heatmaps for confusion matrices of clustering results on the AD-brain dataset, validating the effectiveness of the Exp-Mah metric. Subgraph C investigates the sensitivity of clustering outcomes obtained by the Exp-Mah metric with regard to hyperparameter $\alpha $.

As illustrated in Figure 3, the Exp-Mah metric used in the scHybridBERT method has improved ACC and NMI indexes in single-cell clustering tasks. Confusion matrices obtained by two types of distance metrics also validate the effectiveness and advantage of the Exp-Mah metric. As for the coefficient $\alpha $, the value is suggested to be settled as 0.25 to obtain a balanced trade-off for scRNA-seq datasets including PBMC-Zheng68k and Mouse.

### Employment of graph-structured data

**Figure 4:**
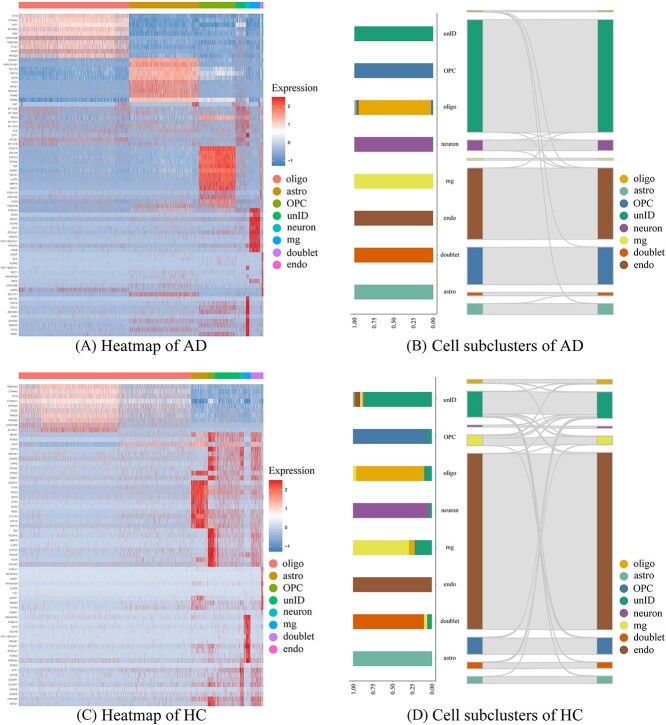
Heatmaps of temporal expression patterns and distributions of cell sub-clusters in the AD-brain case. In **A** and **C**, multiple gene modules govern the expression dynamics of neuronal cells. After single-cell clustering, the stacked bar of Sankey plots in **B** and **D** illustrates the change in the distributions of neuronal cells.

For this Alzheimer’s disease (AD)-brain data, heatmaps of gene expression dynamics are used to demonstrate the temporal patterns underlying single-cell transcriptomics data. Two groups of scRNA-seq datasets, i.e. healthy control (HC) and AD groups, have been analyzed. Meanwhile, Sankey plots of cell sub-clusters are employed to compare the difference between HC and AD group, shown in Figure 4.

As shown by Figure 4A and B, temporal expression patterns of various cell types in the AD group were governed by different groups of marker genes, while the expression patterns in HC group tend to be homogeneous. This phenomenon is consistent with the hypothesis that cell-type-specific marker genes are closely associated with the cellular mechanism of human disease. Meanwhile, Sankey plots of clustering AD-brain data were plotted to validate the feasibility of the scHybridBERT method, yielding an accuracy level of $\sim $0.94.

Distributions of cell subclusters under two situations have been compared. According to the stacked bars in the Sankey plot, confidence levels for cell types including OPC and neuron are higher than other cell types, validating cell heterogeneity. For Alzheimer’s disease, specific neuronal types such as microglia and astrocyte play an essential role in regulating disease progression.

### Distributions of cell sub-clusters

**Figure 5:**
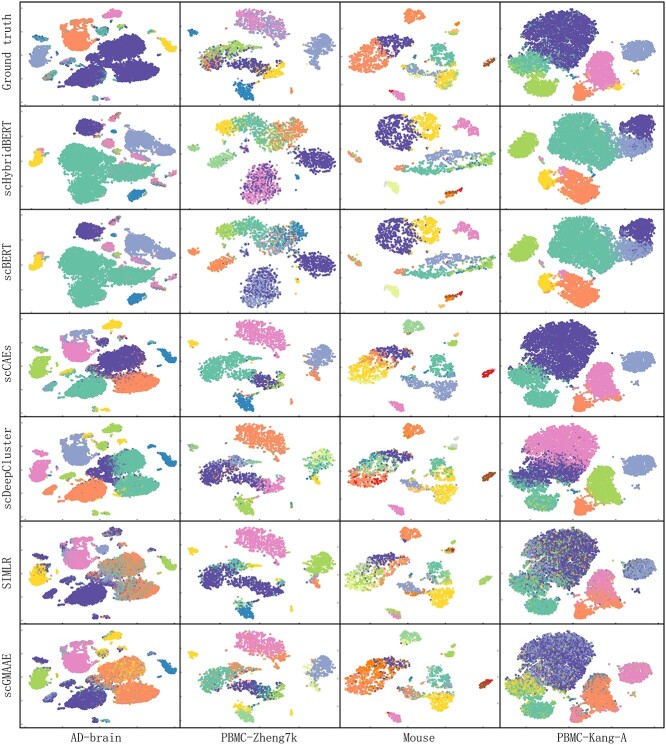
Distributions of cell sub-clusters predicted by scHybridBERT and other SOTA clustering methods. The comparison of t-SNE projections on four datasets illustrates that scHybridBERT architecture better handle the clustering effect of cells, making the cell type detection more consistent with ground truths.

In feature space, distributions of cell sub-clusters are believed to have an association with the differentiation process of specific diseases and the transition of cellular states. In this case, the proportions of cell types have been regarded as useful signal patterns to explore the underlying molecular mechanisms, during disease progression modeling. For benchmark scRNA-seq datasets, cell types were detected by the scHybridBERT architecture and other candidate clustering algorithms. After dimension reduction, the distributions of cell sub-clusters are projected to 2D feature space, shown in Figure 5.

It can be observed from Figure 5 that the cell sub-clusters predicted by the scHybridBERT method have relatively clear boundaries and are well separated in the feature space. However, other clustering approaches face difficulty in distinguishing cell types with similar patterns. It can be found that contours of cell sub-clusters predicted by scHybridBERT and scBERT have shown differences from that of other deep neural networks-based approaches.

### Adaptive MLP-based fusion strategy

This section focuses on the fusion strategy of integrating heterogeneous data modalities including spatiotemporal embedding and cell graphs from multiple perspectives. Temporal embeddings including gene and cell embedding are computed to detect cell-type-specific patterns. Subsequently, spatiotemporal embedding as well as cell graphs are integrated with the multi-view modeling scHybridBERT framework that applies Performer and GATs as backbone models. Both modality-specific components and cross-modality interactions are taken into consideration by the adaptive MLP-based fusion.

Ablation experiments about extracted cell graphs and spatiotemporal embeddings have been conducted to validate the effectiveness of adaptive MLP fusion. Among spatiotemporal embedding, spatial embedding is regarded as graph-structured data that reflect topological features of regulatory systems. Cell clustering experiments have been conducted by multiple combinations of data modalities including spatiotemporal embeddings and cell graphs. Accuracy metrics obtained by multiple combinations of hybrid data modalities have been compared in Table 2.

**Table 2:** Ablation experiments about spatiotemporal embedding and cell graphs in single-cell clustering tasks. Spatial and temporal embedding are combined by the element-wise addition, while spatiotemporal information and cell graphs are fused by adaptive MLP strategy

	PBMC-Zheng7k	PBMC-Kang-A	PBMC-Kang-B	PBMC-Kang-C	Zeisel	AD-brain	Mouse	
Temporal embeddings	0.713	0.926	0.935	0.922	0.946	0.921	0.883	
Cell graph	0.721	0.929	0.939	0.924	0.950	0.925	0.876	
Cell graph+Temporal embedding	0.725	0.932	0.940	0.928	0.952	0.937	0.887	
Cell graph+Spatiotemporal embedding	0.731	0.935	0.945	0.940	0.954	0.940	0.892	

In Table 2, the category of ’Temporal embeddings’ represents conventional gene and expression embeddings that act as inputs of the Performer model, while ’Spatiotemporal embeddings’ denotes the integration of spatial and temporal embedding. Subsequently, spatiotemporal embedding and cell graphs are integrated by adaptive MLP fusion strategy, which considers cross-view interactions. The category of ’Cell graph’ corresponds to clustering outcomes obtained by cell graphs only. It can be observed from Table 2 that spatiotemporal embedding outperforms temporal embedding in cell clustering tasks.

This multi-view scHybridBERT method is able to boost clustering performance by integrating spatial and temporal dynamics of scRNA-seq data. This phenomenon indicates that spatial dynamics at the molecular level play a positive role in the computational analysis of single-cell data.

According to outcomes in Table 3, the adaptive MLP-based fusion strategy outperforms direct concatenation and conducts multi-view modeling with hybrid data modalities. The underlying explanation is that cross-view interactions have been taken into consideration in direct concatenation. Such improvement in clustering accuracy indicates that spatiotemporal embedding and graph-structured data, which were extracted from RNA-sequencing data, could cooperate with each other in capturing deep-level dynamics of omics data.

**Table 3:** Comparison of clustering metrics obtained by the adaptive MLP-based fusion strategy and conventional concatenation in the integration of spatiotemporal embeddings

Strategy	Metrics	PBMC-Zheng7k	PBMC-Kang-A	PBMC-Kang-B	PBMC-Kang-C	Zeisel	AD-brain	Mouse	
Concatenation	ARI	0.562( $\pm $0.002)	0.853($\pm $0.003)	0.919($\pm $0.002)	0.902($\pm $0.002)	0.902($\pm $0.003)	0.932($\pm $0.003)	0.828($\pm $0.003)
	NMI	0.637($\pm $0.002)	0.831($\pm $0.008)	0.864($\pm $0.004)	0.837($\pm $0.004)	0.863($\pm $0.002)	0.837($\pm $0.003)	0.835($\pm $0.004)
	ACC	0.728($\pm $0.002)	0.927($\pm $0.004)	0.942($\pm $0.003)	0.936($\pm $0.003)	0.946($\pm $0.002)	0.933($\pm $0.002)	0.885($\pm $0.002)
Adaptive MLP	ARI	0.574($\pm $0.001)	0.862($\pm $0.001)	0.924($\pm $0.001)	0.907($\pm $0.001)	0.908($\pm $0.004)	0.936($\pm $0.001)	0.837($\pm $0.002)
	NMI	0.643($\pm $0.001)	0.849($\pm $0.003)	0.874($\pm $0.001)	0.841($\pm $0.004)	0.872($\pm $0.005)	0.842($\pm $0.004)	0.843($\pm $0.007)
	ACC	0.734($\pm $0.004)	0.935($\pm $0.001)	0.949($\pm $0.002)	0.940($\pm $0.001)	0.952($\pm $0.001)	0.940($\pm $0.001)	0.892($\pm $0.005)

### Performance evaluation of cell clustering

To validate the effectiveness and advantages of the scHybridBERT framework, clustering accuracy (ACC), normalized mutual information (NMI) as well as adjusted rand index (ARI) were computed to evaluate the clustering performance of various approaches. Multiple scRNA-seq datasets with cell labels were used to verify the accuracy and reliability of deep neural networks-based clustering algorithms, including scBERT, scCAEs [31], scDeepCluster [19], SIMLR [32] and scGMAAE [33].

**Figure 6:**
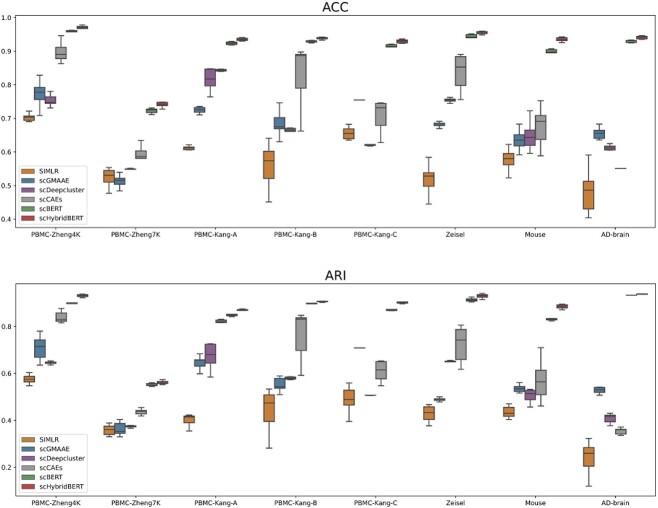
Comparison of evaluation metrics obtained by the scHybridBERT method and other SOTA single-cell clustering algorithms. Replication experiments were conducted to explore the accuracy and robustness of the scHybridBERT method. Evaluation metrics were calculated for 10 replicate experiments.

In Figure 6, the scHybridBERT method has achieved superior clustering accuracy in multiple benchmark datasets and outperformed other SOTA clustering approaches. The advantages of scHybridBERT are not only limited to accuracy but also in robustness, which is reflected by the low variance of clustering outcomes. Deep autoencoder-based models may exhibit relatively high variance in replicate experiments. With 10 experimental replicates, average evaluation metrics of the scHybridBERT method and other SOTA clustering algorithms have been computed and listed in Table 4.

**Table 4:** Performance comparison of the scHybridBERT method and other SOTA clustering approaches with replicated experiments. Average accuracy and deviation metrics are computed to demonstrate the feasibility and robustness of the scHybridBERT method

Datasets	Metrics	SIMLR	scGMAAE	scDeepCluster	scCAEs	scBERT	scHybridBERT
Zeisel	ARI	0.422($\pm $0.045)	0.489($\pm $0.011)	0.651($\pm $0.009)	0.711($\pm $0.094)	0.915($\pm $0.011)	0.927($\pm $0.013)
	NMI	0.614($\pm $0.034)	0.479($\pm $0.067)	0.731($\pm $0.005)	0.719($\pm $0.038)	0.882($\pm $0.012)	0.897($\pm $0.009)
	ACC	0.514($\pm $0.069)	0.615($\pm $0.077)	0.754($\pm $0.009)	0.823($\pm $0.067)	0.946($\pm $0.006)	0.954($\pm $0.004)
Mouse	ARI	0.414($\pm $0.056)	0.529($\pm $0.091)	0.536($\pm $0.079)	0.585($\pm $0.124)	0.833($\pm $0.014)	0.837($\pm $0.002)
	NMI	0.669($\pm $0.031)	0.699($\pm $0.0165)	0.733($\pm $0.015)	0.749($\pm $0.031)	0.843($\pm $0.011)	0.846($\pm $0.007)
	ACC	0.573($\pm $0.049)	0.612($\pm $0.071)	0.659($\pm $0.063)	0.671($\pm $0.082)	0.883($\pm $0.006)	0.892($\pm $0.005)
AD-brain	ARI	0.221($\pm $0.101)	0.513($\pm $0.028)	0.403($\pm $0.026)	0.353($\pm $0.018)	0.933($\pm $0.002)	0.936($\pm $0.001)
	NMI	0.362($\pm $0.015)	0.602($\pm $0.033)	0.596($\pm $0.012)	0.569($\pm $0.015)	0.836($\pm $0.008)	0.842($\pm $0.004)
	ACC	0.497($\pm $0.093)	0.659($\pm $0.023)	0.603($\pm $0.022)	0.544($\pm $0.010)	0.931($\pm $0.002)	0.940($\pm $0.001)
PBMC-Kang-A	ARI	0.341($\pm $0.081)	0.641($\pm $0.043)	0.655($\pm $0.071)	0.824($\pm $0.057)	0.852($\pm $0.010)	0.862($\pm $0.002)
	NMI	0.393($\pm $0.061)	0.655($\pm $0.013)	0.718($\pm $0.018)	0.773($\pm $0.022)	0.845($\pm $0.003)	0.849($\pm $0.003)
	ACC	0.591($\pm $0.031)	0.707($\pm $0.028)	0.806($\pm $0.042)	0.845($\pm $0.044)	0.926($\pm $0.002)	0.935($\pm $0.001)
PBMC-Kang-B	ARI	0.407($\pm $0.126)	0.613($\pm $0.103)	0.616($\pm $0.043)	0.719($\pm $0.128)	0.891($\pm $0.008)	0.924($\pm $0.016)
	NMI	0.501($\pm $0.059)	0.661($\pm $0.035)	0.712($\pm $0.011)	0.761($\pm $0.046)	0.846($\pm $0.002)	0.874($\pm $0.008)
	ACC	0.546($\pm $0.095)	0.694($\pm $0.063)	0.696($\pm $0.058)	0.780($\pm $0.118)	0.935($\pm $0.001)	0.945($\pm $0.004)
PBMC-Kang-C	ARI	0.477($\pm $0.082)	0.679($\pm $0.038)	0.507($\pm $0.002)	0.701($\pm $0.153)	0.875($\pm $0.007)	0.907($\pm $0.002)
	NMI	0.554($\pm $0.034)	0.683($\pm $0.027)	0.661($\pm $0.001)	0.744($\pm $0.05)	0.809($\pm $0.002)	0.841($\pm $0.004)
	ACC	0.617($\pm $0.065)	0.725($\pm $0.005)	0.622($\pm $0.004)	0.763($\pm $0.135)	0.921($\pm $0.004)	0.940($\pm $0.003)
PBMC-Zheng4k	ARI	0.508($\pm $0.095)	0.707($\pm $0.072)	0.644($\pm $0.008)	0.845($\pm $0.031)	0.902($\pm $0.011)	0.925($\pm $0.004)
	NMI	0.616($\pm $0.012)	0.743($\pm $0.026)	0.758($\pm $0.007)	0.833($\pm $0.029)	0.879($\pm $0.019)	0.910($\pm $0.006)
	ACC	0.651($\pm $0.069)	0.769($\pm $0.061)	0.756($\pm $0.025)	0.905($\pm $0.042)	0.962($\pm $0.002)	0.968($\pm $0.002)
PBMC-Zheng7k	ARI	0.316($\pm $0.072)	0.366($\pm $0.037)	0.372($\pm $0.006)	0.432($\pm $0.031)	0.552($\pm $0.007)	0.563($\pm $0.011)
	NMI	0.544($\pm $0.075)	0.581($\pm $0.011)	0.561($\pm $0.005)	0.641($\pm $0.009)	0.623($\pm $0.003)	0.641($\pm $0.008)
	ACC	0.515($\pm $0.038)	0.5118($\pm $0.028)	0.546($\pm $0.005)	0.590($\pm $0.04)	0.713($\pm $0.02)	0.731($\pm $0.004)

It can be found from Table 4 that the scHybridBERT method has outperformed SOTA clustering approaches in multiple scRNA-seq datasets. The robustness of the scHybridBERT method has been validated, shown by low level of deviations and uncertainty in cell type detection. Compared with scBERT, the scHybridBERT architecture has achieved significant improvement in the Zeisel and Mouse cases. Enhanced accuracy of the scHybridBERT method is also observed in AD-brain and multiple PBMC data. This indicates that integrating cell graphs and spatial embedding are able to provide complementary information to temporal dynamics in analyzing single-cell data.

For AD-brain data, deep neural network-based clustering methods including scDeepCluster and scCAEs encountered difficulties in detecting cell subclusters that show similar patterns, leading to low evaluation metrics. Difficulty in detecting specific sub-clusters has also been observed in other groups of scRNA-seq data. Under this circumstance, the scHybridBERT method has provided an efficient solution in analyzing single-cell data, with support of extracted spatial patterns.

## DISCUSSION

In this study, we propose an effective and powerful single-cell clustering method to integrate hybrid data modalities, under the framework of multi-view modeling. To capture spatiotemporal dynamics at the molecular level, cell graphs and gene networks are constructed to obtain multi-view representations of gene sequences. This study proposes an Exp-Mah distance during cell graph construction. The features learned by the performer from gene sequences are combined with the features learned by the graph neural network from cells. Afterward, the aggregation of multi-view information enables the model to have stronger analytical and generalization capabilities.

In this study, the scHybridBERT method integrates hybrid information sources including cell graphs with the support of graph neural networks. This scHybridBERT architecture has shown superior performance in the clustering of scRNA-seq data. In order to employ topological features, two types of graph-structured data have been extracted from a single-cell transcriptomics profile. Cell graphs and spatial embeddings have been extracted from the raw expression matrix as inputs of the GATs and Performer model, respectively.

The first type of graph-structured data is cell graph, which is associated with functional relationships between cells. Another type of graph-structured information used in this study is spatial embedding, which captures inherent interactions between genes. In subsequent training of graph neural networks and Performer models, two types of graph-structured features are integrated with temporal dynamics to boost the model performance. By leveraging both local and global information, scHybridBERT provides a comprehensive description of inter-cellular relationships, by capturing topological features at the molecular level. However, both cell graphs and spatial embedding are single-scale topological information of molecular systems. Future works can investigate the role of multi-scale topological features in the computational analysis of omics data.

Another advantage of scHybridBERT is its high scalability, enabling it to handle large-scale single-cell datasets without the pre-trained model. It is safe to conclude that the scHybridBERT presents effective multi-view modeling in single-cell RNA sequencing data analysis, merging the capabilities of graph neural networks and language models to identify cell types.

The scHybridBERT method developed for single-cell clustering tasks is summarized as follows: (i) spatiotemporal embeddings are extracted from the gene expression matrix and used to integrate gene–gene interactions. Temporal and spatial embedding are fed to the Performer model to detect cell-type-specific patterns; (ii) an exponential Manhattan (Exp-Mah) distance-based measure has been used to construct high-quality cell graphs. In this way, extracted cell graphs capture functional proximity between cells. (iii) The variational AE model was applied to extract low dimensional gene expression for efficient construction of cell graphs while combining these potential gene expressions with graph learning to improve the efficiency of representation learning. (iv) scHybridBERT fully utilizes the advantages of graph neural networks in the employment of graph-structured data. The GAT model aggregates information from node neighbors through attention mechanisms, which have stronger representation ability and flexibility.

Although this scHybridBERT architecture has achieved relatively accurate predictions in cell-type clustering tasks, it still faces certain limitations including the construction of graph-structured data and the fusion of heterogeneous information. In the first case, the issues of non-linearity and sparsity have been frequently discussed in previous analyses of scRNA-seq data. In order to capture static cell–cell interactions, cell graphs are extracted from the gene expression matrix, using the Exp-Mah distance. This exponential combination of correlation coefficients and Manhattan distance has outperformed Euclidean distance. In addition, the formula we use includes the hyper-parameter $\alpha $, which adjusts the weight ratios of two components. Secondly, choosing a variance-based approach in marker gene selection may overlook the important information carried by low or medium-expression genes. Meanwhile, marker genes with important biological functions but low variance may be overlooked. 

During the adaptive MLP-based fusion stage, graph neural networks extract feature vectors from multiple data modalities and perform simple linear combinations. This will face the problems of weight allocation and feature compatibility. Graph neural networks and Performer models extract features from various types of data, which means that their feature representations may have inconsistent properties. Direct concatenation of feature vectors may result in a certain degree of information loss. In future works, we will explore the method to improve the quality of cell graphs and gene–gene interaction, in order to extract deep-level features, and solve the scale problem of feature fusion. In subsequent analysis, we will also integrate graph neural networks into the fine-tuning paradigm to enhance the robustness and model interpretability.

**Key Points**

- In order to conduct multi-view modeling, the scHyBridBERT method diversified the input modalities by extracting spatiotemporal embedding and cell graphs from the single-cell expression data.
- Exp-Mah distance metric was employed to capture cell–cell interactions, which alleviates the drawbacks of Euclidean distance.
- The scHybridBERT architecture employs graph neural networks to mine cell-type-specific patterns from cell graphs, thus boosting the accuracy of single-cell clustering.

## Data Availability

Single-cell transcriptomic datasets with cell-type labels used in experiments have been deposited in the Zenodo platform. All experimental data used in single-cell clustering experiments can be found at https://zenodo.org/record/8256590.

## CODE AVAILABILITY

The source code of this scHybridBERT method, which is used to reproduce experiment outcomes, has been hosted publicly on github, accessible at https://github.com/kaieye/scHybridBERT.
